# A Novel Artificial Hemoglobin Carrier Based on Heulandite-Calcium Mesoporous Aluminosilicate Particles

**DOI:** 10.3390/ijms23137460

**Published:** 2022-07-05

**Authors:** Dino Jordanoski, Damjana Drobne, Neža Repar, Iztok Dogsa, Polona Mrak, Romana Cerc-Korošec, Andrijana Sever Škapin, Peter Nadrah, Natasa Poklar Ulrih

**Affiliations:** 1 Biotechnical Faculty, University of Ljubljana, Jamnikarjeva 101, 1000 Ljubljana, Slovenia; dj6841@student.uni-lj.si (D.J.); damjana.drobne@bf.uni-lj.si (D.D.); neza.repar@bf.uni-lj.si (N.R.); iztok.dogsa@bf.uni-lj.si (I.D.); polona.mrak@bf.uni-lj.si (P.M.); 2 Faculty of Chemistry and Chemical Technology, University of Ljubljana, Večna Pot, 1000 Ljubljana, Slovenia; romana.cerc-korosec@fkkt.uni-lj.si; 3 Slovenian National Bulding and Civil Engineering Institute, Dimičeva Ulica 12, 1000 Ljubljana, Slovenia; andrijana.skapin@zag.si (A.S.Š.); peter.nadrah@zag.si (P.N.)

**Keywords:** silica particles, hemoglobin carrier, encapsulation, mesoporous, spectroscopy

## Abstract

Tetraethyl-orthosilicate (TEOS)-based nanoparticles are most extensively used as a silica-based hemoglobin carrier system. However, TEOS-based nanoparticles induce adverse effects on the hemoglobin structure. Therefore, a heulandite-calcium-based carrier was investigated as a novel silica-based hemoglobin carrier system. The heulandite-calcium mesoporous aluminosilicate particles (MSPs) were fabricated by a patented tribo-mechanical activation process, according to the manufacturer, and its structure was assessed by X-ray diffraction analysis. Upon hemoglobin encapsulation, alternation in the secondary and tertiary structure was observed. The hemoglobin-particle interactions do not cause heme degradation or decreased activity. Once encapsulated inside the particle pores, the hemoglobin shows increased thermal stability, and higher loading capacity per gram of particles (by a factor of >1.4) when compared to TEOS-based nanoparticles. Futhermore, we introduced a PEGlyted lipid bilayer which significantly decreases the premature hemoglobin release and increases the colloidal stability. The newly developed hemoglobin carrier shows no cytotoxicity to human umbilical vein endothelial cells (HUVEC).

## 1. Introduction

With the increased interest in nanobiology and nanoparticle research [1,2,3], there have been many studies in recent years on the use of nanocarriers, such as inorganic nanomaterials, as loading structures for hemoglobin (Hb). Tu et al. suggested the use of tetraethyl-orthosilicate-based nanoparticles as a hemoglobin carrier system [4]. However, synthesized tetraethyl-orthosilicate-based (TEOS) nanoparticles have adverse effects on Hb structure and lymphocyte cell lines [5]. Therefore, significant changes in the protein structure can become an issue. To reduce these concerns, we developed a novel hemoglobin carrier based on heulandite-calcium (heulandite-Ca) mesoporous aluminosilicate particles (MSPs). Heulandite-Ca MSPs are crystalline inorganic polymers that are based on a three-dimensional arrangement of SiO_4_ and AlO_4_ tetrahedra that are connected through their oxygen atoms to form large negatively charged lattices with Bronsted and Lewis acid sites [6].

Upon Hb binding and encapsulation inside the particle pores, the heulandite-Ca MSPs induce partial denaturation to the Hb protein structure. The secondary and tertiary structure of the Hb was examined by various spectroscopic techniques (Ultraviolet-visible and fluorescence spectrometry, Circular dichroism, Fourier Transform Infrared Spectrometry) and its preserved function by performing a peroxidase-like activity assay. Once encapsulated, the Hb is more thermally stable, and hence the MSPs provide a safe and protective microenvironment. Using thermogravimetric analysis, we achieved higher hemoglobin loading capacity when compared to the TEOS nanoparticles (by a factor of >1.4).

Only three key steps were required to assemble the hemoglobin carrier: isolation and collection of the MSPs; Hb encapsulation; and coating with a PEGylated lipid bilayer (LB). The spontaneous coating of the Hb-loaded heulandite-Ca MSPs (Hb-MSPs) provided significantly increased colloidal stability and decreased the premature release of hemoglobin, therefore reducing the content of free Hb which is proven to be toxic [7]. Confocal microscopy imaging was used to visualize lipid bilayer coated Hb-MSPs (LB-Hb-MSPs). Therefore, our newly developed hemoglobin carrier shows great promise as a carrier, but not as an oxygen carrier, due to the ferrous state of the iron in the hemoglobin, which cannot bind to oxygen.

## 2. Results and Discussion

### 2.1. Morphology and Mesoporous Structure of the MSPs

**Figure 1:**
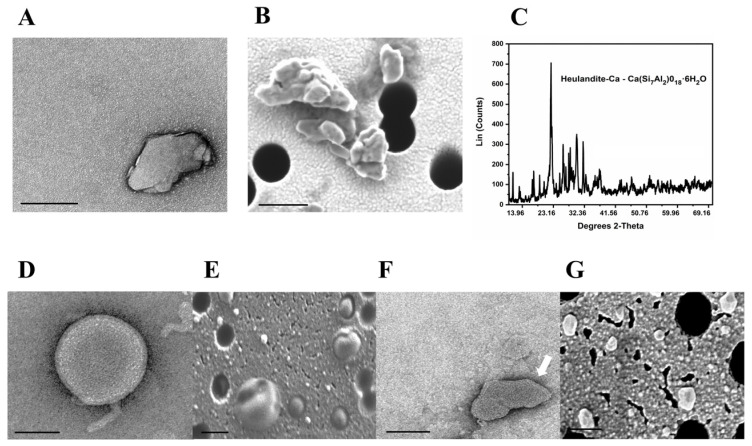

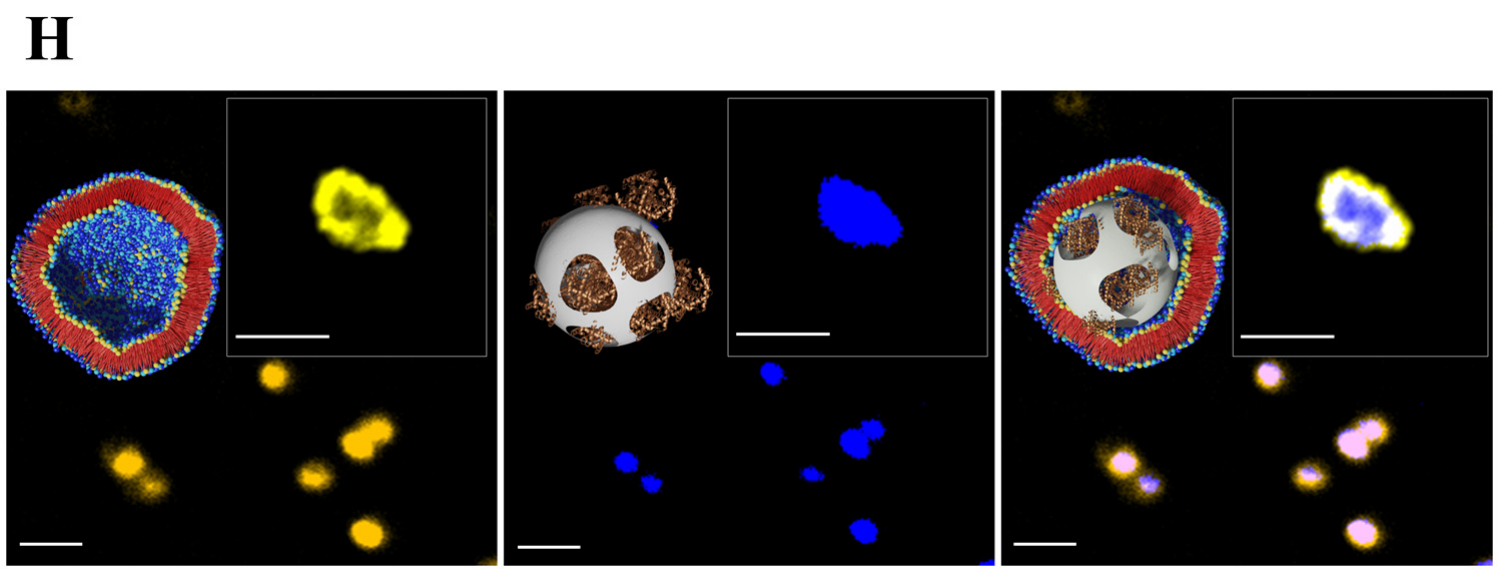
Part I: (**A**) A representative transmission electron microscopy (TEM) image of MSPs (Heulandite-Calcium Mesoporous Aluminosilicate Particles). (**B**) Representative scanning electron microscopy (SEM) image of MSPs. (**C**) X-ray diffraction pattern of the MSPs. (**D**,**E**) Representative TEM and SEM images of empty liposomes, retrospectively. (**F**,**G**) Representative TEM and SEM images of LB-MSPs (lipid bilayer coated Heulandite-Calcium Mesoporous Aluminosilicate Particles) (white arrow: showing the lipid layers around the particle). Scale bar for all images, 100 nm. Part II: (**H**) Representative confocal images of a DOPE-LR labeled liposome (yellow color), a FITC-labeled Hb-MSP (purple color), and Hb-loaded LB-MSPs (purple color with yellow outside layer). Scale bar, 2 µm. Inserts: Representative confocal images of DOPE-LR labeled liposome, FITC-labeled Hb-MSP and Hb-loaded LB-MSPs. Scale bar, 1 µm.

**Figure 2:**
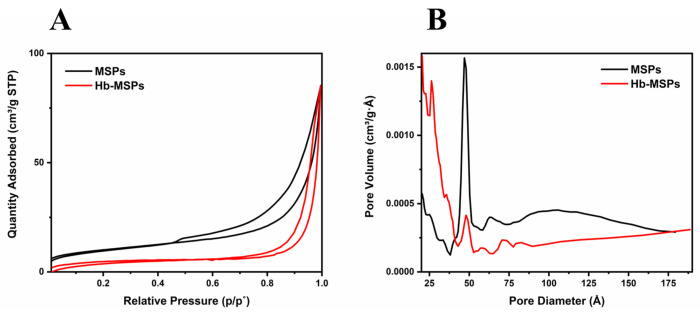
(**A**) Nitrogen adsorption-desorption isotherms of MSPs and Hb-MSPs. (**B**) Plots of pore diameter vs. pore volume, as calculated from the desorption isotherms using the Barrett–Joyner–Helenda model.

The morphology and mesoporous structure of the MSPs were determined by TEM and SEM. From the TEM and SEM images, the MSPs were seen to be nonspherical, with up to 500 nm for their largest dimension (Figure 1A,B). The crystalline structure and the chemical composition of these heulandite-Ca MSPs were determined using XRD analysis (Figure 1C). Furthermore, the crystal structure of heulandite-Ca is shown in (Supplementary Figure S3), as visualized using the VESTA software, with the atom coordinates from the American Mineralogist Crystal Structure Database [8,9]. To characterize the mesoporosity and channels within the MSPs, and to demonstrate the encapsulation of Hb, nitrogen sorption measurements were performed. Both MSPs and Hb-MSPs showed characteristic type IV isotherms with type H3 hysteresis (Figure 2A). The Brunauer–Emmet–Teller model surface area of MSPs and Hb-MSPs were 34 m^3^/g and 28 m^3^/g, respectively. MSPs showed a narrow pore-size distribution, with a peak at 47 Å and a pore volume below 209 Å of 0.068 cm^3^/g. The pore volume of Hb-MSPs was reduced to 0.048 cm^3^/g for the same range of pores, and the peak at 47 Å was greatly reduced. Data from the desorption curves are shown in Figure 2B.

Using confocal microscopy, we additionally confirmed successful Hb-MSPs encapsulation inside the lipid bilayer of the liposomes (Figure 1H). In addition, we examined and compared the shape and size of empty liposomes using TEM and SEM electron microscopy (Figure 1D–E) and lipid-bilayer coated MSPs (Figure 1F,G). Our results suggest and support our claim of successfully coating the MSPs into the liposome bilayer, and, as seen from the images the lipid-bilayer coated MSPs, take the shape of the MSPs, while the empty liposomes are spherical in shape. The XRD pattern of the lipid-bilayer coated MSPs does not show the characteristic XRD peaks of MSPs, which again confirms the successful coating around the particles (Supplementary Figure S1). These data were supported by the field emission SEM energy dispersive spectroscopy analysis (Supplementary Figure S2).

### 2.2. Determination of MSPs Hemoglobin-Loading Capacity

**Figure 3:**
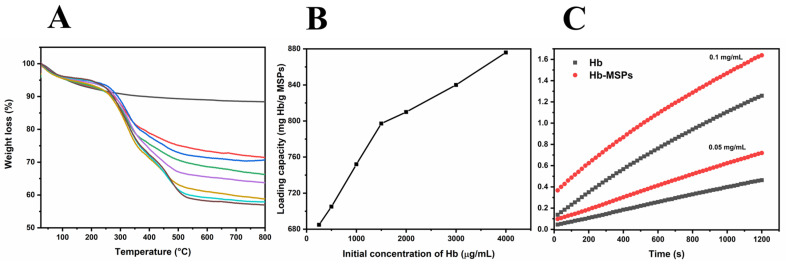
(**A**) Thermogravimetric analysis curves of Hb-loaded MSPs with different initial Hb concentrations (0, 0.25, 0.5, 1, 1.5, 2, 3, 4 mg/mL; top to bottom). (**B**) Corresponding loading capacities of Hb into MSPs calculated by thermogravimetric analysis. (**C**) ABTS catalyzed by native Hb and Hb-loaded MSPs. The enzymatic activity of Hb was measured at 418 nm as the catalytic conversion of oxidation of ABTS.

Thermogravimetric analysis is one of the most frequently used methods to determine the loading capacity of a substance on inorganic nanoparticles [10,11]. Upon heating to 800 °C, the samples underwent total weight loss, and the weight loss up to 100 °C was a result of the thermo-desorbed water on the particles (Figure 3A). The total weight loss (W) that corresponded to the loaded Hb was correlated with the initial Hb concentration.

With an initial Hb concentration of 4000 µg/mL, the maximum Hb-loading capacity was 876 mg/g (Figure 3B), which is greater than the Hb-loading capacity reported by Tu et al. for their TEOS nanoparticles (621 mg/g) [4]. In summary, we achieved higher loading capacity using MSPs with a lower surface area (by a factor of >1.4), which gives promising opportunities for future surface modifications for even higher Hb encapsulation.

Furthermore, to investigate the loading capacity in greater detail, we evaluated the Hb loading at lower initial concentrations of Hb (400–700 µg/mL) and at one concentration of MSPs (0.5 mg/mL) (Supplementary Figure S4). This revealed a linear correlation between Hb concentration and the amount of encapsulated Hb, as confirmed by the high value of R^2^ (0.994). It is interesting to note that this correlation was lost at higher Hb concentrations, presumably due to pore blockage of the MSPs with Hb.

### 2.3. Enzyme-like Activity of Encapsulated Hb Compared to Free Hb

Hemoglobin is a peroxidase-like protein, meaning that the heme prosthetic group can successfully catalyze the reduction of hydrogen peroxide with high substrate and reactive efficiency. We thus conducted an enzyme-like activity test. The goal was to determine the peroxidase-like activity of encapsulated Hb compared to free Hb, to provide information on the Hb activity that remains once it is encapsulated. Therefore, the oxidation of ABTS by hydrogen peroxidase was used as an indicator [12]. Here, the encapsulated Hb showed a higher peroxidase-like activity compared to free Hb. As predicted, higher concentrations of Hb resulted in faster conversation of hydrogen peroxide (Figure 3C). The greater activity can be attributed to the crowded microenvironment in the nanopores of the MSPs [13]. It has also been shown that lysozyme has higher catalytic activity when encapsulated in matching-sized nanopores [14], as close to the lysozyme dimensions. The present study therefore sheds light on the important role of the macromolecular crowding effect in Hb immobilization, and provides a new approach for creating more efficient and stable enzyme-immobilized systems for biomedical purposes [15].

### 2.4. Spectroscopic Analysis of Free and Encapsulated Hemoglobin, and Lipid-Bilayer-Coated MSPs

#### 2.4.1. Ultraviolet-Visible Spectroscopy

The folding of the Hb protein was investigated by inspection of the Soret band in the UV-Vis absorption spectrum of free Hb and Hb-MSPs, as Hb is sensitive to changes in pH, its microenvironment, and oxidative stress [16]. The Hb-MSP absorbance curves in the Soret band showed a slightly broadened peak, due to light scattering by the MSPs. A good linear relationship between absorbance (405 nm) and Hb-MSPs was obtained (R^2^ = 0.985), which was similar to native Hb (R^2^ = 0.995). This confirms that partial unfolding may have occurred upon binding (Supplementary Figure S5). 

**Figure 4:**
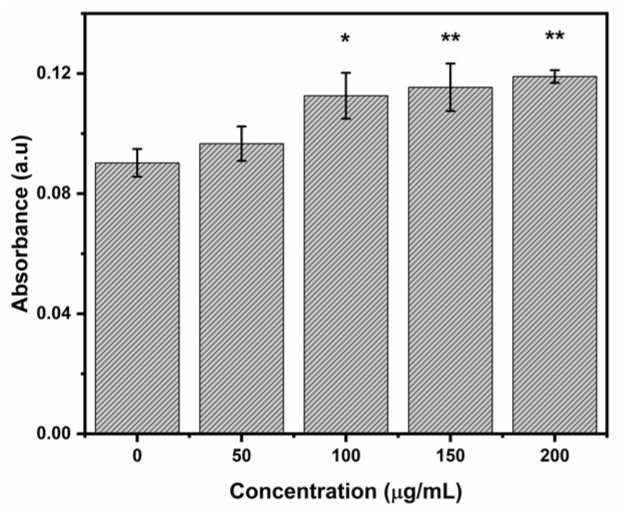
Iron release from hemoglobin in the presence of increasing concentrations of MSPs. Data are means ±SD of two independent experiments. * *p*-value < 0.05, ** *p*-value < 0.01 vs. control preforming one way analysis of variance (ANOVA) by Fisher’s comparison test.

We also asked whether the Hb and MSP interactions resulted in iron release from the Hb. For the ferrozine assay, Hb (300 µg/mL) was incubated with different concentrations of MSPs (0, 50, 100, 180, 360 µg/mL). Here, the Hb binding significantly induced iron release from the Hb only at the higher MSP concentrations (Figure 4) [5]. Also, recent studies have shown increased release of free iron from Hb upon binding to (TEOS)-based nanoparticles at much lower particle concentrations, which can lead to destructive changes to the Hb molecule, and therefore these MSPs represent a better substitute [5,17].

#### 2.4.2. Fluorescence Spectrometry

**Figure 5:**
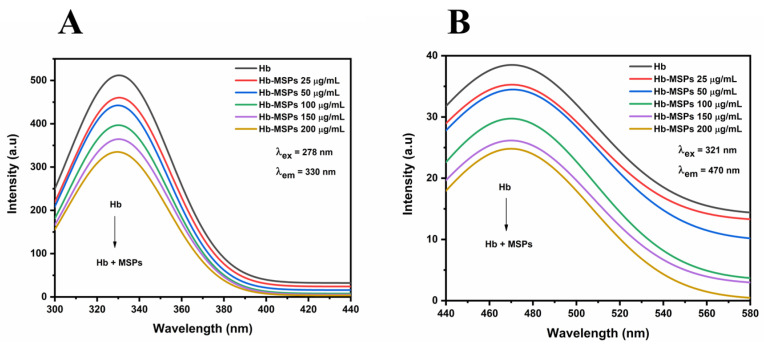
(**A**) Steady-state fluorescence spectra of Hb and Hb-MSPs at 278 nm excitation wavelength. (**B**) Heme degradation study of Hb-MSPs at 321 nm excitation wavelength.

Fluorescence emission spectroscopy is the most sensitive method for studying molecular interactions in solutions, and therefore we investigated the Hb binding and interactions with the MSPs. Excitation of Hb at 278 nm stimulates fluorescence of its tryptophan residues at about 330 nm. As shown in Figure 5A, with an increase in MSP concentration, there was a decrease in Hb fluorescence intensity, but the emission maximum did not shift to shorter or longer wavelengths. These results indicated that there is a strong interaction between the Hb molecules and the MSPs, followed by a partial denaturation. Azimipour et al. showed that using TEOS-based nanoparticles does indeed cause structural changes to Hb, with an increase in fluorescence intensity, as the binding induces the production of heme degradation products and reactive oxygen species [5].

On the other hand, heme groups do not show any intrinsic fluorescence intensity, while heme degradation produces highly fluorescent products [5,18]. The fluorescence emission spectra were scanned from 440 nm to 580 nm with an excitation wavelength of 321 nm. As shown in Figure 5B, there was fluorescence quenching for the Hb fluorescence intensity due to its binding onto the MSPs without any shift in the maximum emission peak, which indicated that the MSPs do not induce heme degradation upon Hb binding.

#### 2.4.3. Circular Dichroism

**Figure 6:**
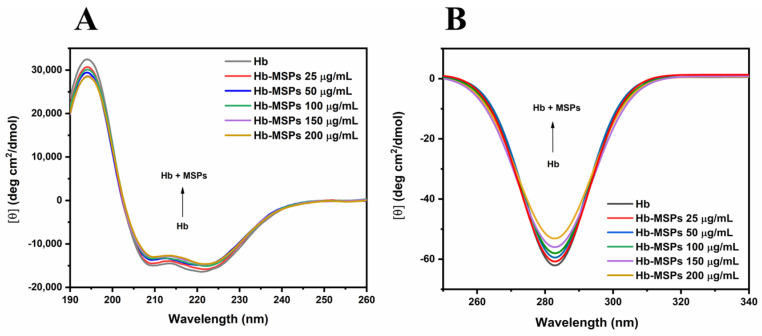
Far−UV (**A**) and near-UV (**B**) circular dichroism spectra of Hb and Hb−MSPs at different MSP concentrations (25, 50, 100, 150, 200 µg/mL; bottom to top) and at Hb concentrations of 0.1 mg/mL (**A**) and 1 mg/mL (**B**).

Circular dichroism is a sophisticated spectroscopy method that is used mainly to determine secondary structures of proteins [19], and also for characterizing protein–ligand interactions and structural disorder [20]. When residues appear in a repeating pattern, as occurs in secondary structures, their peptide bonds produce typical far-UV CD spectra (190–260 nm), thus revealing their presence. As shown in Figure 6A, free Hb and bound Hb spectra are presented. Hb has a predominantly α-helix secondary structure. The CD spectrum showed two minima at 208 nm and 222 nm, which are characteristic of α-helical structures (Figure 6A).

The overall CD signal decreases with the increase in the MSPs concentrations, thus suggesting that the hemoglobin appears to get partially denatured with a loss in its secondary structure. In the near-UV region (250–340 nm), aromatic amino acids affect the CD signals, thus providing information about the tertiary structure of a protein. Hb has a characteristic near-UV CD spectrum below 300 nm, with a minimum at ~285 nm, due to the aromatic amino acids. Figure 6B shows the near-UV CD spectrum of Hb and Hb-MSPs for different MSPs concentrations. Here, upon Hb binding to the MSPs, the CD signal decreases with the increase in the MSPs concentration, proving that the hemoglobin gets partially denatured with loss in its tertiary structure. These data shows that the Hb binds to the MASPs, and additionally supported by our fluorescence study (Figure 5A). Upon release from the MSPs, the hemoglobin shows to be partially denatured with loss in its α-helical structure (Supplementary Figure S6). On the other hand, using TEOS-based nanoparticles as carriers has been shown to induce greater differences in the CD signals with a significant shift between the different treatment groups, thus indicating that greater changes occur to the Hb upon binding to TEOS-based nanoparticles [5].

#### 2.4.4. Fourier Transform Infrared Spectrometry

Fourier transform infrared spectroscopy is a non-destructive method for structural characterization of proteins and polypeptides. The IR spectra are interpreted in terms of vibrations of structural repeats. The repeat units in proteins can give rise to nine characteristic IR absorbance bands (amide A, B, I–VII).

**Figure 7:**
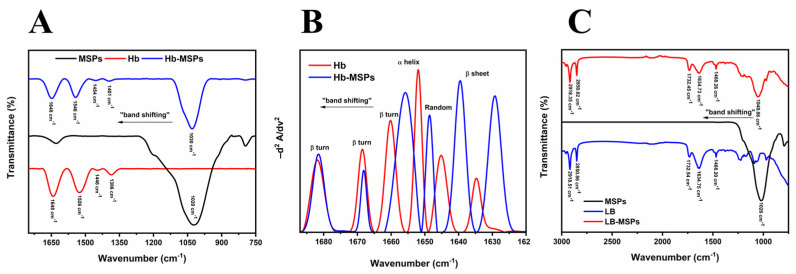
(**A**,**C**) Fourier transform infrared spectra of heulandite−Ca MSPs (**A**,**C**), and free Hb and Hb−loaded MSPs (**A**), and empty liposomes and liposome−encapsulated MSPs (**C**). (**B**) Curve−fitting of the inverted second−derivate spectra of the amide I region of Hb and Hb−loaded MSPs.

Here, we investigated the amide I band in greater detail (1690–1620 cm^−1^), as well as the amide II and amide III bands. These bands are the most often used for determination of protein structure. Amide I and amide II are the two major bands in the Hb IR spectrum (Figure 7A). The most sensitive region is amide I, and, hence, the second-derivate and curve-fitting function indicated the secondary structure of Hb (Figure 7B). The amide I band originates from C=O stretching vibration of the amide group, together with bending of the N–H bond and stretching of the C–N bond [21,22,23]. The amide II band results from N–H bending (as 40–60% of the potential energy) and C–N stretching vibration (18%-40%). The amide III band is caused by the different side chains and prosthetic groups of a protein, and it is thus different for each protein. As shown in Figure 7A for Hb, the peak at 1020 cm^−1^ corresponds to Si–O stretching (1000–1100 cm^−1^) of the aluminosilicate lattice, and the peak at 793 cm^−1^ (800–200 cm^−1^) is a result of Al–O in the structure of the MSPs, thus showing that these are indeed aluminosilicate-based particles [24,25].

The Hb-MSPs spectra showed band shifting for all of these amide regions, including for the Si–O group region, which indicted the interactions and binding of Hb onto the MSPs as new bonds were formed [26]. Furthermore, the second-derivate of the IR Hb-MSPs spectrum (Figure 7B) shows how the random coil and β-sheet/turn signal predominates in comparison to free Hb, indicating that the hemoglobin is partially denatured upon binding which is supported by our CD results (Figure 6A). This study confirms the successful binding of Hb onto the MSPs and also confirms the aluminosilicate structure of the MSPs.

The successful lipid coating by liposomes was further ascertained by these FTIR studies (Figure 7C). The characteristic peaks for liposomes were observed at 2900–2850 cm^−1^ and 1732 cm^−1^, which correspond to the stretching vibrations of the alkyl groups and ester groups, respectively [27]. The same peaks were observed in the LB-MSP spectra, thus showing successful liposome coating around the MSPs [28]. Also, band shifting in the Si–O group region was seen (from 1020–1049 cm^−1^), which resulted from the liposome–MSP interactions [29].

### 2.5. Differential Scanning Calorimetry Analysis of Free and Encapuslated Hemoglobin, and Lipid-Bilayer-Coated MSPs

**Figure 8:**
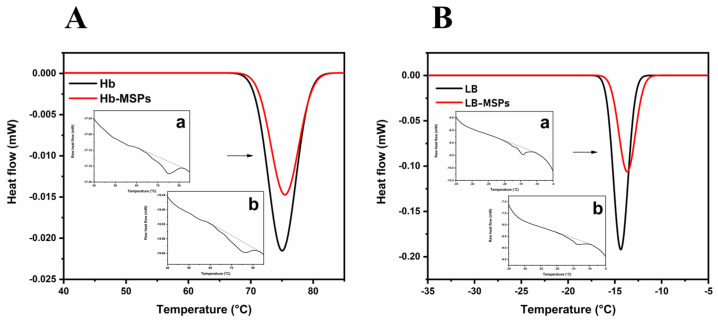
(**A**) A typical differential scanning calorimetry (DSC) thermal denaturation profile for free Hb and Hb−loaded MSPs. Insets (a, b): raw DSC spectra for free Hb (a) and Hb−loaded MSPs (b). (**B**) DSC thermograms of empty liposomes and lipid−bilayer-coated MSPs. Insets (a, b): raw DSC spectrum for empty liposomes (a) and liposome−encapsulated MSPs (b). Heating rate, +10 °C/min.

To study the thermal stability of the encapsulated Hb and the liposome-encapsulated MSPs, we conducted a differential scanning calorimetry analysis. Figure 8A shows that encapsulated Hb induced alternations in the position of the denaturation peak towards higher temperatures (Table 1), compared to free Hb. This is due to the nature of the interactions between Hb and the MSPs, as well as to the Hb encapsulation within the nanopores of the MSPs, as the MSPs provide a protective microenvironment for Hb upon its binding [30,31]. Also, this shows that bound Hb is a little more stable in comparison to free Hb, as this provides a stable environment at lower temperatures. The LB-MSPs showed a small shift in the main transition peak (*T_m_*) to higher temperatures, thus indicating increased membrane rigidity (Figure 8B) [32,33], which correlates with the membrane fluidity analysis (see below).

As the MSPs bind to the membrane surface, they penetrate below the polar headgroups of the lipids, and hence make the membrane less fluid, which increases the transition temperature peak to higher temperatures.

### 2.6. Membrane Fluidity Assay

**Figure 9:**
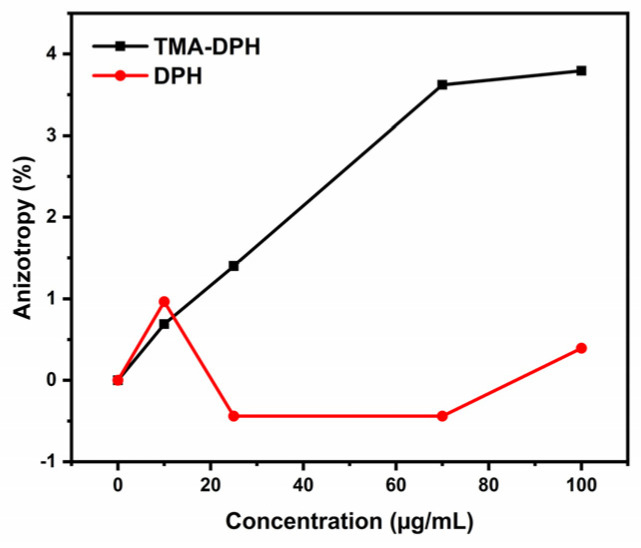
Changes in anisotropy of the membrane probes (TMA−DPH and DPH).

Furthermore, we studied the interactions between the liposomes and the MSPs, and the MSP effects on membrane fluidity. For the effects on lipid membrane fluidity, DOPE:DOPC:PEG liposomes were used, and the recordings were carried out using two different membrane probes: TMA-DPH and DPH (see Materials and Methods). Figure 9 shows that the anisotropy change in the TMA-DPH probe increases towards positive values, thus indicating a decrease in membrane fluidity [32];

However, no significant changes in anisotropy were seen for the DPH probe. These results indicate that the MSPs appear to be embedded on the membrane surface of the liposomes, with interactions with the polar headgroups of the lipids. This will result in an increase in the order of the lipids [32] that did not reach the hydrophobic core of the lipid bilayer (Supplementary Figure S7).

### 2.7. Colloidal Stability and Release Profiles of Hemoglobin from Heulandite-Ca MSPs and Lipid-Bilayer-Coated MSPs

**Figure 10:**
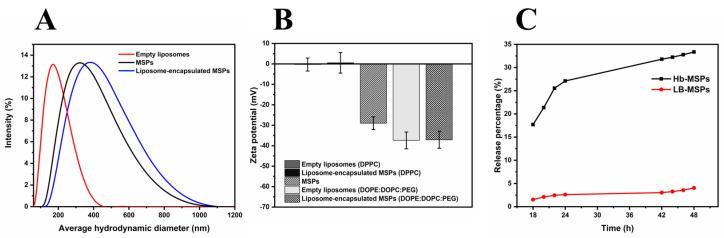
(**A**) Dynamic light scattering for the mean hydrodynamic diameters of empty liposomes, MSPs, and liposome−encapsulated MSPs (LB−MSPs) (in 1 mM phosphate buffer, pH 7.4). (**B**) Zeta potentials before and after MSP encapsulation in the liposomes. The error bars represent the zeta potential data from the streaming potential measurements. (**C**) Release profiles of Hb−loaded MSPs and LB−Hb−MSPs (in phosphate−buffered saline, 37 °C, pH 7.4). The error bars represent the standard deviation.

The long-term colloidal stability of the LB-Hb-MSPs is important for future biomedical applications. Therefore, we used a lipid bilayer (liposomes) for the Hb-MSPs. After the Hb-MSPs were coated with the lipid membrane, the mean hydrodynamic diameter increased from 320 nm to 390 nm, which indicated the liposome encapsulation of the MSPs (Figure 10A). The mean hydrodynamic diameter remained stable for at least 4 days (Supplementary Figure S8).

Next, we measured the average Zeta potential of the MSPs before and after encapsulation, to once again confirm their successful encapsulation by the liposomes. Two types of liposomes were used here: DPPC and DOPE:DOPC:PEG. The DPPC liposomes had a neutral Zeta potential (−1.8 ± 3.2 mV), and were therefore not stable in suspension, while the DOPE:DOPC:PEG liposomes were negatively charged (−37.4 ± 4.1 mV). As the MSPs are negatively charged (−29 ± 3.1 mV), after encapsulation with the neutral DPPC liposomes, the LB-MSP Zeta potential remained neutral. Similarly for the DOPE:DOPC:PEG liposomes, where the LB-MSP Zeta potential remained negative, thus indicating successful encapsulation of the MSPs within the liposomes (Figure 10B) (Table 2).

Next, the cumulative release of Hb from MSPs and LB-Hb-MSPs was investigated in vitro (Figure 10C). Hb-MSPs showed a burst release during the first 24 h, with the release of 27.1% of the loaded Hb, while for LB-Hb-MSPs this was decreased to 2.6%. After 48 h, the cumulative releases of Hb from Hb-MSPs and LB-Hb-MSPs were 33.4% and 4%, respectively. This shows that the lipid bilayer acts as a physical barrier to lower the release of Hb from the MSPs (Figure 10C).

### 2.8. Citotoxicity of Heulandite-Ca MSPs and Lipid-Bilayer-Coated MSPs

**Figure 11:**
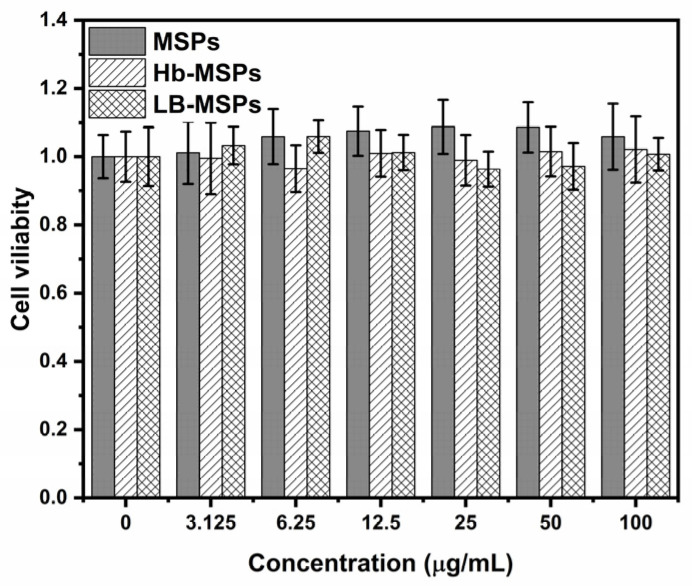
Cell viability after 24 h exposure to increasing concentrations of Hb, Hb−loaded MSPs (Hb−MSPs), and liposome-encapsulated MSPs (LB−MSPs). No significant differences were seen vs. control preforming one way analysis of variance (ANOVA) by Fisher’s comparison test.

These heulandite-Ca MSPs and LB-MSPs were not cytotoxic against the HUVEC cell line at concentrations upwards to 100 µg/mL (Figure 11). Since our membrane fluidity assay results show interaction of the MSPs with only the polar heads of the membrane and not the hydrophobic parts, we predicted that the particles could not penetrate the membrane. Many reports suggest that smaller size particles are more cytotoxic then the larger one [34,35]. This might be because smaller particles can penetrate the cell membrane and cause cellular damage [36]. Literature reports that TEOS-based nanoparticles were cytotoxic to lymphocytes at 10 µg/mL, with >20% reduction in cell viability [5]. One reason for low cytotoxicity could be the size of MSPs, which is in the range of 276 ± 129 nm, as well as possible agglomeration in the cell medium. In order to support our prediction and membrane fluidity results, we performed a cytotoxicity assay on the HUVEC cell line to prove the concept.

## 3. Materials and Methods

### 3.1. Materials

Bovine Hb (Mw 64.5 kDa, Methemoglobin), 2′,2′-azino-bis (3-ethylbenzothiazoline-6-sulfonic) acid (ABTS), and fluorescein isothiocyanate (FITC) were from Sigma-Aldrich (St. Louis, MI, USA), and were used as received. 1,2-Dioleoyl-*sn*-glycero-3-phosphocholine(DOPC),1,2-dioleoyl-*sn*-glycero-3-phosphoethanolamine(DOPE),1,2-dioleoyl-*sn*-glycero-3-phosphoethanolamine-N-[methoxy(polyethyleneglycol)-2000] (ammonium salt) (PEG2000-PE), DOPE N-(lissamine rhodamine B sulfonyl) (ammonium salt) (DOPE-LR) and dipalmitoyl phosphatidylcholine (DPPC) were from Avanti Polar Lipids (Alabaster, AL, USA). The composition of the phosphate-buffered saline (PBS) was: 14.99 mM K_2_HPO_4_, 5 mM KH_2_PO_4_, 150.07 mM NaCl, for an ionic strength of 270 mM, at pH 7.4. The phosphate buffer (PB) was prepared by mixing 1 mM Na_2_HPO_4_ and 1 mM NaH_2_PO_4_ at a molar ratio of 5:2, for an ionic strength of 1 mM. Milli-Q water was used throughout. The MSPs in a form of powder, were bought from LUCERN VIP DOO, (Skopje, North Macedonia).

### 3.2. Preparation of Heulandite-Ca MSP Suspension

The heulandite-Ca MSP suspension was obtained by dispersing the powder in 1 mM PB (pH 7.4) at 20 mg/mL. The suspension was shaken for 2 min using a vortex mixer (Vortex 3; IKA, Staufen, Germany), followed by 20 min sonification in a water bath (ultrasonic cleaner UT8031/EUK; Shesto, Watford, UK). The MSP suspension was then centrifuged for 3 min at 2000 RCF (Rotanta 460R; Hettich, Buford, GA, USA), with immediate collection of the supernatant. The MSP suspension was stored at room temperature and sonicated for 5 min before use.

### 3.3. Loading Hemoglobin into Heulandite-Ca MSPs

The dispersed heulandite-Ca MSPs at 2 mg/mL in 1 mM PB (pH 7.4) were mixed with a series of 400–700 µg/mL bovine Hb solutions, for a final MSP concentration of 0.5 mg/mL, with shaking for 10 min (ThermoShaker; 400 rpm, 25 °C; Biometra TS1 ThermoShaker; Analytik Jena, Jena, Germany). For further physical characterization, the Hb-MSPs were collected by centrifugation and washed twice (13,700 RCF, 5 min; centrifuge 5425 R; Eppendorf, Germany), and the nonencapsulated Hb in the supernatant was quantified using a plate reader (Safire 2; Tecan, Männedorf, Switzerland).

### 3.4. Preparation of the Lipid Film

The lipid film was prepared by dispensing stock solutions of 25 mg/mL DOPC (80 µL), 25 mg/mL DOPE (40 µL), and 25 mg/mL PEG2000-PE (30 µL) into a rotary evaporator flask. All of the lipids were dissolved in chloroform-methanol (7:3, *v*/*v*). A lipid film was formed by slow evaporation of the chloroform-methanol in the flask over 3 h, to constant weight. The film was kept at 4 °C until further use. Fluorescent lipids (DOPE-LR) were incorporated into the lipid mixture at 1 wt% for the fluorescence analyses.

### 3.5. Preparation of Lipid-Bilayer-Coated Hb-MSPs

For coating of the Hb-MSPs, the previously prepared lipid film was hydrated to form large unilamelar vesicles, for 45 min with 1 mM PB (pH 7.4) without (empty liposomes) and with Hb-MSPs (loaded liposomes). During the hydration at 50 °C (empty liposomes) and 37 °C (loaded liposomes) the lipid film was sonicated for 30 s every 15 min, followed by a final 3 min sonification (ultrasonic cleaner UT8031/EUK; Shesto, Watford, UK). At the end, the loaded liposomes were collected with centrifugation and washed twice (20,000 RCF, 10 min; centrifuge 5425 R; Eppendorf, Germany).

### 3.6. Structure and Shape of Heulandite-Ca MSPs, and LB-MSPs

The morphology and mesostructure of the MSPs were characterized using scanning electron microscopy (SEM) and transmission electron microscopy (TEM). SEM imaging was conducted using a field-emission electron microscope (Ultra Plus; Carl Zeiss, Jena, Germany) with an accelerating voltage of 1 kV. Samples for SEM imaging were deposited onto a gold membrane and sputter-coated prior to analysis. For TEM, ~4 µL of the liposome suspension was applied to the Formvar-coated 400-mesh copper TEM grid for 30 s. Before applying the sample, the grids were coated with an ~4 nm thick carbon layer and glow discharged (ACE200 vacuum coater; Leica, Munich, Germany). Samples were stained with 1% aqueous uranyl acetate and examined using a transmission electron microscope (CM100; Philips, Amsterdam, The Netherlands). Images were recorded with a CCD camera (Orius SC 200; Gatan, Pleasanton, CA, USA) using the Digital Micrograph software. X-ray diffraction (XRD) analysis (AXS D4 Endeavor; Bruker, Billerica, MA, USA) was carried out with Cu Kα radiation (λ = 0.154 nm) and a Sol-X detector. A scanning step of 0.02° and a counting time of 6 s per step were used for the measurements. The hydrodynamic size distribution, polydispersity index, and zeta-potential were measured using dynamic light scattering (ZetaSizer Nano ZSP; Malvern Instruments, Malvern, UK).

### 3.7. Labeling of Hemoglobin with Fluorescein Isothiocyanate

Hemoglobin (10 mg) was dissolved in 5 mL 100 mM sodium carbonate (pH 9). FITC was dissolved in dimethylsulfoxide at 1 mg/mL, and 0.25 mL of FITC solution was added to the Hb solution. The mixture was stirred overnight at 4 °C. The resulting FITC-labeled Hb was purified by size exclusion chromatography using a Sephadex-G25 column and PBS as the eluent.

### 3.8. Confocal Microscopy Imaging

Fluorescence images of the liposomes were obtained under fluorescence microscopy (CLSM microscope; Axiovsion Z1, LSM800; Zeiss, Jena, Germany) using the plan-apochromat 100×/1.40NA oil objective. The CLSM images were acquired using two laser channels: 488 nm laser at 4% intensity, with the detector (GaAsP PMT, Thorlabs, Newton, NJ, USA) gain set to 850 V to acquire fluorescence from FITC-labeled Hb; and 561 nm laser at 4% intensity, with the detector gain set to 700 V for rhodamine-B-labeled DOPE. The emitted fluorescence was recorded at 410 nm to 540 nm and at 540 nm to 700 nm for the two channels, respectively. The pinhole size was set to 40 µm. The images acquired had a pixel size of 0.067 µm × 0.067 µm × 0.250 µm, with 16-bit color depth. To improve the signal-to-noise ratio, 16 frames were averaged for each image, with the pixel dwell time set to 2.05 µs. The Zen 2.3 software (Carl Zeiss, Jena, Germany) was used to acquire the images, and ImageJ [37] was used for image visualization.

### 3.9. Surface Area and Mesoporosity of Heulandite-Ca MSPs, and Hb-MSPs

Nitrogen absorption–desorption isotherms were obtained using a surface area analyzer (ASAP 2020; Micromeritics, Norcross, GA, USA). Before the measurements, the MSP samples were degassed for 2 h at 0.033 mbar and 105 °C, and the Hb-MSP samples for 12 h at 0.033 mbar and 25 °C. The specific surface areas were calculated from the absorption data in the low-pressure range according to the Brunauer–Emmet–Teller model [38]. The pore size distribution was determined following the Barrett–Joyner–Helenda model [39].

### 3.10. Loading Capacity of Hemoglobin into Heulandite-Ca MSPs

For the thermogravimetric analysis (TGA/DSC1; Mettler Toledo, Ljubljana, Slovenia) the samples were placed into 150 µL platinum crucibles and heated from 25 °C to 800 °C at a heating rate of 10 K/min. During the measurements, the furnace was purged with air with a flow rate of 50 mL/min. The blank curve was subtracted. The maximum loading capacity (mg/g) for Hb was obtained according to [4] and [11], by mixing the MSP suspensions and Hb at higher initial concentrations (0, 0.25, 0.5, 1, 1.5, 2, 3, 4 mg/mL). The mass concentrations of the solutions were determined as follows: 100 μL aluminum crucibles were weighed together with their lid (MX5 balance; Mettler Toledo, Ljubljana, Slovenia). Hb-MSPs were pipetted into the crucible using a 50 μL pipette, quickly covered with the aluminum lid, and hermetically pressed to prevent evaporation. After pressing, the crucible was weighed again, and the mass of the solution was calculated from the difference. The lid was then carefully pierced, and the samples were dried to constant weight (3 days at room temperature). The crucibles with the dried samples were weighed again to determine the mass of the solid residue. The proportion (%) of the total loaded Hb was determined and calculated according to Equations (1) and (2); [4,20].

(1) $$\frac{H_{1}-L_{1}}{100-H_{1}}=\frac{H_{2}-W-L_{2}}{100-H_{2}},$$

(2) $$W=H_{2}-L_{2}-\frac{\left(H_{1}-L_{1}\right)\left(100-H_{2}\right)}{100-H_{1}},$$

where *H*_1_ and *H*_2_ are the the total weight loss up to 800 °C of the MSPs and Hb-MSPs, respectively, and *L*_1_ and *L*_2_ are the weight loss up to 100 °C due to the thermo-desorbed water for the MSPs and Hb-MSPs, respectively.

### 3.11. Peroxidase-like Activity of Hemoglobin-Loaded MSPs

The peroxidase-like activity of Hb after encapsulation in the MSPs was measured using ABTS; [13]. The ABTS solution was prepared by dissolving 15 mg ABTS in 1 mL MilliQ water and 9 mL acetic acid. Hydrogen peroxide (30% [*w*/*w*] in water; 1 mL) was diluted into 30 mL MilliQ water, Hb (0.05, 0.1 mg/mL; 5 µL, PBS) and Hb-MSPs (0.05, 0.1 mg/mL; 5 µL, PBS) were mixed with 150 µL hydrogen peroxide in 96-well plates, followed by immediate addition of 45 µL ABTS solution. Absorbance of oxidized blue-green ABTS˙+ was monitored at 418 nm every 20 s for 20 min using a plate reader (Safire 2; Tecan, Männedorf, Switzerland). The controls were performed using hemoglobin-free PBS and MSPs suspended in PBS (0.05, 0.1 mg/mL). The measurements were carried out in duplicate. The MSPs concentration was the same as the Hb concentration (0.05, 0.1 mg/mL) throughout this experiment. At the end, the blank measurements (hemoglobin-free PBS, MSPs suspened in PBS) were subtracted from the sample measurements to avoid unwanted scattering. In addition, we subtracted the baseline of Hb to make sure that no signal was interfering from the Hb.

### 3.12. Free Iron Assay

Iron release upon Hb binding to MSPs was investigated using a ferrozine assay, as described previously [5]. After Hb loading (0.3 mg/mL) using different concentrations of MSPs (0, 50, 100, 180, 360 µg/mL), 100 µL 100 mM ascorbic acid was added, and the solution was incubated for 5 min at room temperature. Then, 50 µL 16% ammonium acetate and 50 µL 16 mM ferrozine were added to the samples, which were mixed well for 5 min at room temperature. Then, the absorbance was measured at 562 nm using a plate reader (Safire 2; Tecan, Männedorf, Switzerland). The measurements were carried out in duplicate.

### 3.13. Spectroscopic Analysis of Free and Encapsulated Hemoglobin, and Lipid-Bilayer-Coated Hb-MSPs

#### 3.13.1. Ultraviolet-Visible Spectrometry

The UV-VIS analysis of free Hb and Hb-MSPs with different initial concentrations of Hb (25, 50, 100, 150, 200, 250, 300, 350 µg/mL) was evaluated in the range from 300 nm to 600 nm. The absorbance maximum peak of Hb was measured at 405 nm (8453 UV-VIS spectrophotometer; Hewlett Packard, Palo Alto, CA, USA). The MSP concentration was kept constant at 250 µg/mL. Hb-free 1 mM PB and 250 µg/mL MSP suspensions were used as controls. The measurements were carried out in duplicate.

#### 3.13.2. Fluorescence Spectrometry

A steady-state fluorescence spectrometry (Cary Eclipse; Varian, Agilent Technologies, Santa Clara, CA, USA) was used to follow the changes in the Trp environment of free Hb and Hb-MSPs. An excitation wavelength of 278 nm was used, and the fluorescence emission spectra were recorded at a wavelength range from 300 nm to 440 nm. The heme degradation study was followed at an excitation wavelength of 321 nm, in the wavelength range from 440 nm to 580 nm. The final Hb concentration was 0.3 mg/mL for all of the samples. Upon the addition of different concentrations of MSPs (25, 50, 100, 150, 200 µg/mL), the spectra were recorded. Hb-free PB and MSPs at different concentrations in PB were used as controls. The measurements were carried out in duplicate.

#### 3.13.3. Circular Dichroism

The changes in the secondary and tertiary structures of Hb upon addition of different concentrations of MSPs (25, 50, 100, 150, 200 µg/mL) were followed using circular dichroism (CD; J-1500 circular polarimeter; Jasco, USA). The CD spectra were measured with a 0.1-cm path length quartz cell in the far-UV CD region (190–260 nm), and with a 1-cm path length quartz cell in the near-UV CD region (250–340 nm). The Hb concentration for the far-UV CD analysis was 0.1 mg/mL, and for the near-UV CD analysis, 1 mg/mL. Hb-free PB and MSPs at different concentrations in PB were used as controls.

#### 3.13.4. Fourier Transform Infrared Spectrometry

For Fourier transform infrared spectrometry (FTIR) analysis (Frontier FT-IR; PerkinElmer, Waltham, MA, USA) of MSPs, the sample was in powder form, while the concentration of free Hb and Hb-MSP solutions was 10 mg/mL. A constant temperature of 25 °C was maintained by a temperature controller (GladiATR; Pike Technologies Inc., Madison, WI, USA). The experiment was conducted according to the literature [27]. A higher Hb concentration from 3 mg/mL to 20 mg/mL was required to obtain better spectral resolution and lower signal-to-noise ratio. FTIR spectra were collected in the wavenumber range from 1700 cm^−1^ to 750 cm^−^^1^, at a resolution of 4 cm^−1^ at 25 °C. Before the measurements, the Hb and Hb-MSP solutions were prepared fresh, following a protein purity check using SDS-PAGE electrophoresis, then determination of the Hb concentrations. After collecting the background spectrum, buffer spectrum, and sample spectrum, buffer subtraction was carried out. The results are also plotted as the second derivate of absorbance as a function of wavenumbers in the range from 1700 cm^−1^ to 1620 cm^−1^ for secondary structure analysis of Hb and Hb-MSPs, using the OriginLab software, version 95E. The empty liposomes and Hb-MSP-loaded liposomes spectra were scanned in the wavenumber range from 3000 cm^−1^ to 900 cm^−1^. Before the measurements, the suspensions were centrifugated for 10 min at 13,700 RCF, to obtain the empty liposomes and the Hb-MSP-loaded liposomes. Then, the FTIR analysis was carried out for the pellets obtained.

### 3.14. Differential Scanning Calorimetry Analysis of Free and Encapsulated Hemoglobin, and Lipid-Bilayer-Coated MSPs

For the thermal denaturation profiles, temperature-modulated differential scanning calorimetry measurements were performed (DSC 2500; TA Instruments, New Castle, DE, USA). For each measurement, the Hb sample (27.21 mg; 10 mg/mL) and the Hb-MSP sample (28.86 mg; Hb, 10 mg/mL; MSPs, 20 mg/mL) were placed in a hermetic aluminum pan. An empty hermetic aluminum pan was used as reference. Hb-free PB and MSPs in PB (20 mg/mL) were used as controls. To investigate the liposome-MSP (LB-MSP) interactions, empty liposome sample (29.34 mg; 10 mg/mL), and LB-MSP sample (29.34 mg; 10 mg/mL) were placed in a hermetic aluminum pan. Liposome-free PB and MSPs in PB (10 mg/mL) were used as controls. The measurements were carried out in duplicate.

### 3.15. Membrane Fluidity Assay

To investigate the MSP–membrane interactions, membrane fluidity assays were carried out using two membrane probes: 1,6-diphenylhexatriene (DPH) and its trimethylammonium derivative (TMA-DPH). Anisotropy values were measured on a fluorescence spectrophotometer (Cary Eclipse; Agilent Technologies, Inc., Santa Clara, CA, USA) for the empty liposomes (100 µg/mL) and the liposomes in the presence of different MSP concentrations (10, 25, 70, 100 µg/mL). After the lipid film was hydrated, ultrasonication (Q500 Sonicator, QSonica, Newtown, CT, USA) followed for 30 min to obtain small unilamellar vesicles (liposomes) to study the membrane interactions. The measurements were carried out at an excitation wavelength of 358 nm and an emission wavelength of 410 nm. For 2.5 mL liposomes and liposomes loaded with MSPs (lipid-bilayer-coated [LB]-MSPs) working suspension, the fluorescent probes were added as 2.5 µL 2 mM TMA-DPH and 1 mM DPH. The measurements were carried out in duplicate.

### 3.16. Release Profiles of Hemoglobin from Heulandite-Ca MSPs and Lipid-Bilayer-Coated MSPs

The in-vitro release profiles of Hb from the Hb-MSPs and LB-Hb-MSPs were investigated by suspension of Hb-MSPs and LB-Hb-MSPs in PBS (at 37 °C, pH 7.4) at 1 mg/mL. The solutions were incubated at 37 °C using a shaker (400 rpm; Biometra TS1 ThermoShaker; Analytik Jena, Germany). At various times, the solution was centrifuged (13,700 RCF, 5 min, centrifuge 5425 R; Eppendorf, Germany), and the supernatants were replaced with fresh PBS. The released Hb in the supernatant was determined using a plate reader (Safire 2; Tecan, Männedorf, Switzerland). The measurements were carried out in duplicate.

### 3.17. Cytotoxicity Assay of Heulandite-Ca MSPs and Lipid-Bilayer-Coated MSPs

Human umbilical vein endothelial (HUVEC) cells were cultured in Dulbecco’s modified Eagle’s medium supplemented with 10% (*v*/*v*) fetal bovine serum and 4 mM L-glutamine. The cells were cultured in a humidified atmosphere of 5% CO_2_, 95% air at 37 °C. The cells were routinely sub-cultured twice a week (at ~2 × 10^4^ cells/cm^2^). Before harvesting the cells (TrypLE Select; Thermo Fisher Scientific, Waltham, MA, USA), they were washed with PBS (without Ca^2+^ and Mg^2+^). The cells were resuspended in the cell medium, centrifuged at 200× *g* for 5 min, and seeded equally into three 96-well plates at a seeding density of 2 × 10^4^ cells/cm^2^ (i.e., 6666 cells/well), as 100 µL/well. After a 24-h incubation in a controlled atmosphere, the medium was discarded and 100 µL of the final MSP samples diluted in the cell medium (Dulbecco’s modified Eagle’s medium supplemented with 10% [*v*/*v*] fetal bovine serum and 4 mM L-glutamine) was added to the wells. After 24-h exposure to the MSP samples, CyQuant reagent was added directly to the cells (100 µL/well). The cells were incubated with this detection reagent for 60 min at 37 °C. Fluorescence intensity at the bottom of the wells was measured at 480 nm and 535 nm (excitation/emission) using a standard plate reader.

### 3.18. Statistical Analysis

The analysis of the data was performed using one way analysis of variance (ANOVA) by Fisher’s comparison test. All data were reported to be statistically significant at * *p*-Value < 0.05, ** *p*-Value < 0.01 vs. control.

## 4. Conclusions

In summary, we present here a novel and successful artificial hemoglobin carrier based on natural heulandite-Ca MSPs. The most widely used TEOS-based carriers have been shown to have adverse effects on Hb structure [5]. TEOS nanoparticles might thus represent a less favourable option for biological, medical, and food applications due to the cytotoxicity shown towards biological systems. They induce Hb displacement and structural changes for Hb degradation, while the heulandite-Ca MSPs only induce partial denaturation [5], as summarized in (Supplementary Table S1). These heulandite-Ca MSPs provide a safe and protective microenvironment for the Hb molecule, followed by a partial denaturation of the hemoglobin molecule. Upon Hb binding and its release, the redox activity was preserved. This is shown schematically in (Supplementary Figure S9). We introduced a lipid bilayer coating (as liposomes) for the Hb-MSPs to increase the colloid stability and to prevent the premature release of Hb. Our newly developed carrier shows greatly decreased hemoglobin cumulative release in comparison to TEOS nanoparticles, 4% and 20%, retrospectively, which gives a better property since free Hb is toxic [7]. This newly designed carrier did not show cytotoxicity to HUVEC cells at concentrations up to 100 µg/mL. The PEGylated Hb-MSPs only interact with the hydrophilic polar headgroups of the membranes, and not with the inner hydrophobic tails of the membrane lipids. The studied Heulandite-Calcium Mesoporous Aluminosilicate Particles show far better properties than conventional TEOS nanoparticles, which holds great potential for development of hemoglobin loading and delivery systems for biomedical purposes. In order to broaden the scope of possible future applications of our particles, the cytotoxicity and cellular intake should be investigated with more adequate cell lines, such as blood cells or hepatic cells. 

## Figures and Tables

**Table 1:** Thermodynamic profiles of the denaturation peaks of Hb and Hb-loaded MSPs, and phase transitions of liposomes and liposomes incubated with MSPs (in 1 mM phosphate buffer, pH 7.4).

Sample	*T_m_* (°C)
Hb	75.72 ± 0.9
Hb-loaded MSPs	76.73 ± 0.5
Empty liposomes	−14.08 ± 0.1
Liposome-encapsulated MSPs	−13.07 ± 0.4

Data are means ± SD.

**Table 2:** Mean hydrodynamic diameters for MSPs, Hb-loaded MSPs, and liposome-encapsulated MSPs (in 1 mM phosphate buffer, pH 7.4), and their Zeta potentials.

Sample	Hydrodynamic Diameter (nm)	Polydispersity Index	Zeta Potential (mV)
MSPs	276 ± 129	0.168	−26.7 ± 5.38
Hb-loaded MSPs	283 ± 148	0.215	−19.1 ± 4.05
Liposome-encapsulated MSPs	292 ± 224	0.368	−36.8 ± 4.66

Data are means ± SD.

## Data Availability

Not applicable.

## Supplementary Materials

The following supporting information can be downloaded at: https://www.mdpi.com/article/10.3390/ijms23137460/s1.
