# Supplementary material for: A Novel Artificial Hemoglobin Carrier Based on Heulandite-Calcium Mesoporous Aluminosilicate Particles
Source: Int J Mol Sci. 2022 Jul 5;23(13):7460. doi: 10.3390/ijms23137460 (PMC9267069; doi:10.3390/ijms23137460)
Supplement: Supplementary file 1 [file ijms-23-07460-s001.zip › ijms-1777896-supplementary.pdf]

Supplementary Material

A Novel Artificial Hemoglobin Carrier Based on Heulandite-Calcium Mesoporous Aluminosilicate Particles

Dino Jordanoski [1], Damjana Drobne [1], Neža Repar [1], Iztok Dogša [1], Polona Mrak [1], Romana Cerc-Korošec [2], Andrijana Sever Škapin [3], Peter Nadrah [3], Nataša Poklar Ulrih [1*]

[1] University of Ljubljana, Biotechnical Faculty, Jamnikarjeva 101, 1000 Ljubljana, Slovenia; Dino Jordanoski: dj6841@student.uni-lj.si. Damjana Drobne: damjana.drobne@bf.uni-lj.si. Neža Repar: neza.repar@bf.uni-lj.si. Iztok Dogša: iztok.dogsa@bf.uni-lj.si. Polona Mrak: polona.mrak@bf.uni-lj.si. Nataša Poklar Ulrih: natasa.poklar@bf.uni-lj.si.

[2] University of Ljubljana, Faculty of Chemistry and Chemical Technology, Večna pot, 1000 Ljubljana, Slovenia; Romana Cerc-Korošec: romana.cerc-korosec@fkkt.uni-lj.si.

[3] Slovenian National Bulding and Civil Engineering institute, Dimičeva ulica 12, 1000 Ljubljana, Slovenia; Andrijana Sever Škapin: andrijana.skapin@zag.si. Peter Nadrah: peter.nadrah@zag.si.

* Correspondence: Nataša Poklar Ulrih: natasa.poklar@bf.uni-lj.si; Tel.: (+386 1 3203 780, 100 Ljubljana, Slovenia)

A

B

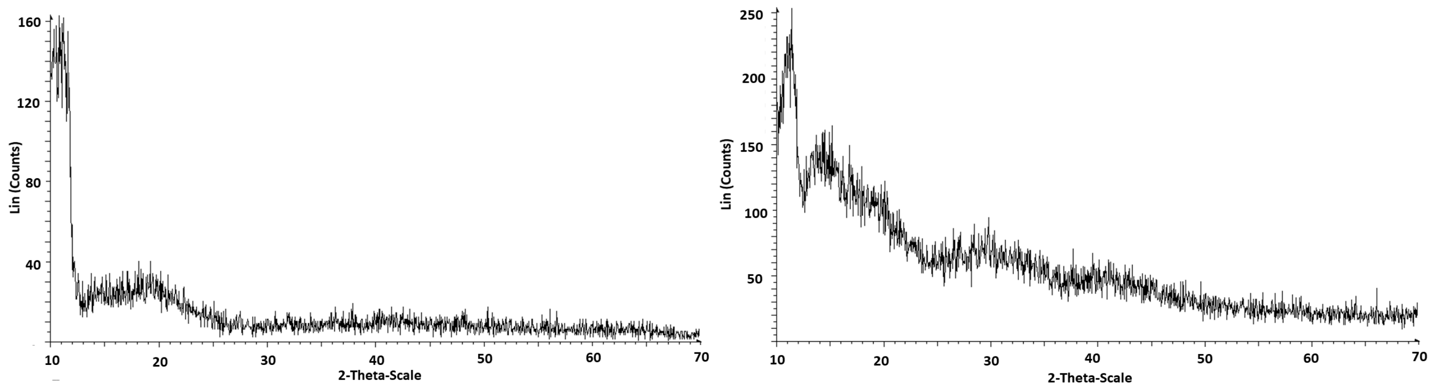

Figure S1. X-ray diffraction patterns of empty liposomes (LB) (**A**) and liposome-encapsulated MSPs (LB-MSPs)(**B**).

A

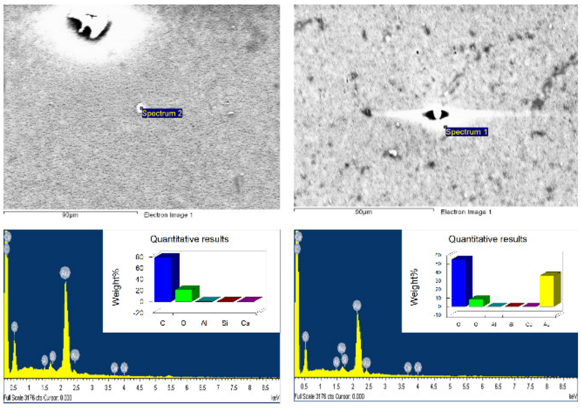

Figure S2. Field emission SEM energy dispersive spectroscopy analysis of empty liposomes (**A**) and liposome-encapsulated MSPs (**B**).

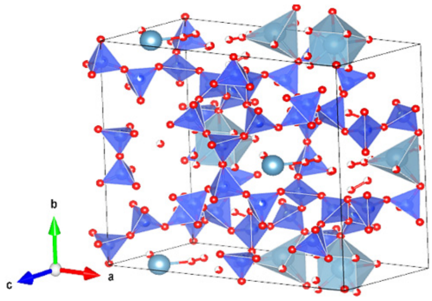

Figure S3. Crystal structure of heulandite-Ca, visualized using the VESTA software, with atom coordinates from the American Mineralogist Crystal Structure Database (VESTA, n.d.).

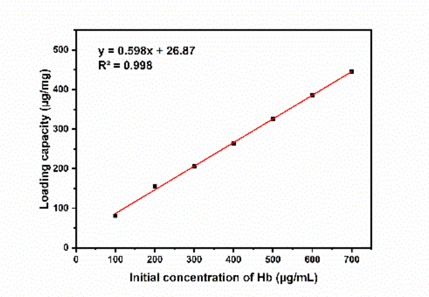

Figure S4. Hemoglobin-loading capacity for MSPs at low-loading concentrations, using a plate reader (Safire 2; Tecan; absorbance, 405 nm; 400-700 µg/mL).

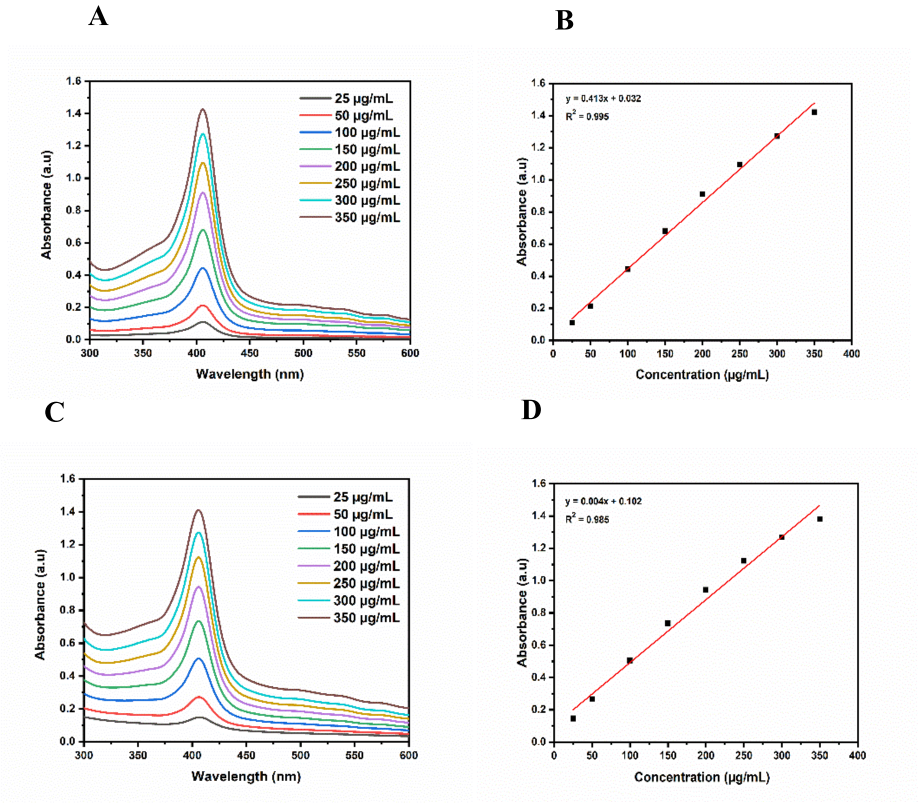

Figure S5. (A, C) UV-VIS absorbance spectra of Hb (25-350 µg/mL; 1 mM PB as control) **(A)** and Hb-loaded MSPs (25-350 µg/mL; 250 µg/mL MSPs in 1 mM PB as

control) (**C**). (**B, D**) Standard curves for absorbance (405 nm) of Hb (**B**) and Hb-loaded MSPs (**D**).

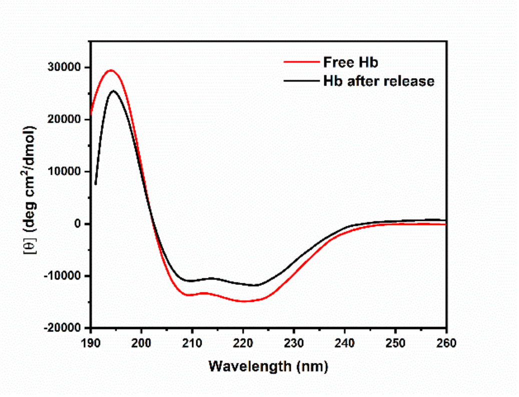

Figure S6. Far-UV circular dichroism spectra of free Hb and Hb after release from Hb-loaded MSPs.

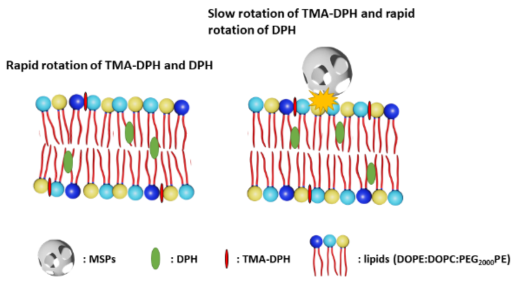

Figure S7. Schematic illustration of the MSP–liposome membrane interactions. DPH, 1,6-diphenylhexatriene; TMA-DPH, DPH trimethylammonium derivative; DOPE, 1,2-dioleoyl-*sn*-glycero-3-phosphoethanolamine; DOPC, 1,2-dioleoyl-*sn*-glycero-3-phosphocholine; PEG, polyethylene glycol.

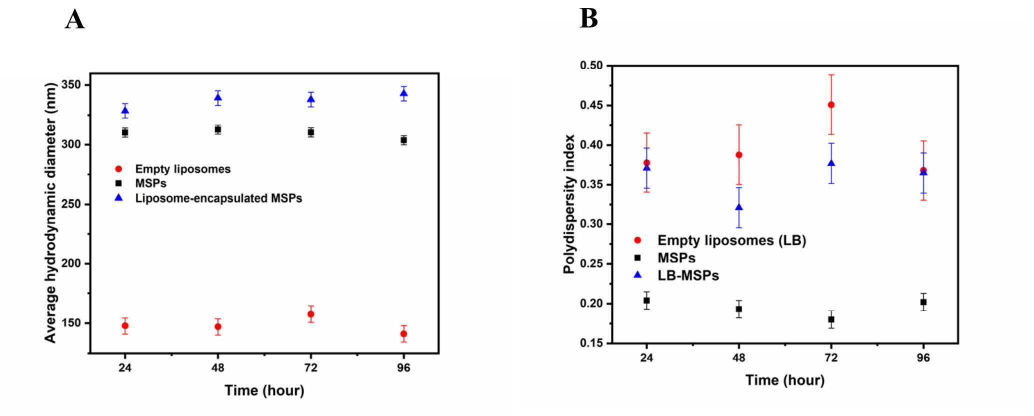

Fig. S8. (A) Size stability of LB, MSPs and LB-MSPs); (B) Coresponding PDI values.

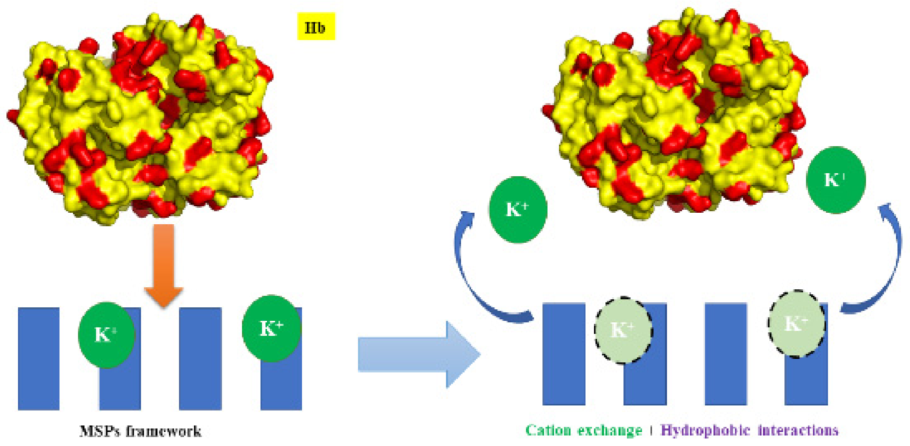

Figure S9. Schematic diagram of adsorption of hemoglobin (Protein) onto natural zeolite (heulandite-Ca). The hydrophobic surface of hemoglobin was modeled with the PyMOL software, version 2.4.1. Yellow, Hb backbone; red, Hb positively charged amino acids (i.e., Arg, His, Lys).

Table S1. Summary of hemoglobin effects upon interaction with tetraethyl orthosilicate (TEOS)-based particles and heulandite-Ca MSPs.

**Analysis technique**	**Particle type**	
	**TEOS-based particles**	**Heulandite-Ca MSPs**
**Fourier transform infrared spectroscopy**	Structural changes induced	Partial denaturation and successful binding
**Fluorescence spectroscopy**	Degradation induced	Partial denaturation and successful binding
**Circular dichroism**	Displacement and denaturation	Partial denaturation
**UV-Vis spectroscopy**	Iron release induced at low particle concentrations	Iron release induced at high particle concentrations (≥100 µg/mL)
**Peroxidase-like activity**	Preserved redox activity of bound hemoglobin	Higher redox activity of bound hemoglobin

**Cytotoxicity**	Cytotoxic at low concentrations (≥10 μg/mL)	No cytotoxicity up to ≥100 μg/mL
